# Happiness and hypertension prevalence: A global analysis

**DOI:** 10.1371/journal.pgph.0006472

**Published:** 2026-06-03

**Authors:** Moosa Tatar, Amir Habibdoust, Soheila Farokhi, Mohammad Reza Faraji, José A. Pagán, Xing Song

**Affiliations:** 1 Department of Pharmaceutical Health Outcomes and Policy, University of Houston, Houston, Texas, United States of America; 2 Institute for Data Science and Informatics, University of Missouri-Columbia, Columbia, Missouri, United States of America; 3 Department of Computer Science, Utah State University, Salt Lake City, Utah, United States of America; 4 Strive Health, Denver, Colorado, United States of America; 5 Department of Public Health Policy and Management, School of Global Public Health, New York University, New York, New York, United States of America; 6 Department of Biomedical Informatics, Biostatistics, and Medical Epidemiology, University of Missouri-Columbia, Columbia, Missouri, United States of America; Augusta University, UNITED STATES OF AMERICA

## Abstract

The prevalence of hypertension around the world is high while hypertension control is relatively low. The objective of this study is to investigate the association between happiness and hypertension prevalence across countries. We used World Happiness Report (WHR) data, NCD (non-communicable disease) Risk Factor Collaboration (NCD-RisC) data, and machine learning methods (K-means clustering, XGBoost) to assess the influence of key variables that may explain variations in national happiness scores on predicting hypertension prevalence for males and females in 151 countries with complete concurrent data across all global regions for the year 2019. The K-means clustering method resulted in four clusters of countries based on the happiness features. Countries in groups with higher happiness scores had a relatively lower prevalence of Hypertension. The XGBOOST analysis showed that GDP per capita was the most important feature predicting the prevalence of hypertension for both males and females. Also, generosity and life expectancy were other important features predicting hypertension for males. Healthy life expectancy, social support, and freedom to make life choices were important features predicting hypertension for females. Social support and healthy life expectancy were stronger predictors of hypertension prevalence in males, whereas healthy life expectancy and GDP per capita were most influential for females. Sex-specific public health considerations may be valuable for better understanding patterns of hypertension prevalence worldwide. Multi-faceted, integrated policy approaches that target not only economic factors but also consider a broader societal well-being may help inform efforts to address hypertension across countries.

## Introduction

Hypertension is a chronic health condition that is associated with cardiovascular disease, chronic kidney disease, disability, and mortality [1–4]. Approximately one-third of adults aged 30–79 (1·3 billion individuals) worldwide has been diagnosed with hypertension (WHO, 2023) [5] and, of those with hypertension, only 23% of treated females and 18% of treated males achieve targeted blood pressure levels [6]. Also, in 2019, approximately half of all cardiovascular deaths were attributable to high blood pressure [7]. In addition, 62% of deaths from chronic kidney disease were attributed to high blood pressure [5] This translates to 18% of male deaths and 20% of female deaths [6]. Currently, hypertension takes the lives of approximately 10 million people every year [8]. Global healthcare costs of suboptimal blood pressure control account for approximately 10% of the world’s total healthcare expenditures [9]

The connection between health behaviors such as diet, exercise, smoking, drinking, and blood pressure is well-documented [10–13]. Emerging research is focusing on psychosocial factors that may be related to hypertension [14–16]. Higher optimism levels are associated with reduced hypertension risk [14], and chronic psychosocial stress is associated with an increased risk of hypertension [16]. Among psychosocial factors, a number of studies have focused on the association between happiness or subjective well-being and blood pressure [10]. Individuals with higher levels of subjective happiness and emotional vitality seem to experience lower rates of hypertension [17–19]. Similarly, subjective happiness has been found to have a negative correlation with hypertension, suggesting that happiness could play a significant role in preventing hypertension [20].

Despite these findings, most existing research focuses on individual-level analyses, examining personal risk factors for hypertension while disregarding broader societal influences. Some studies have explored the relationship between national happiness and hypertension [21,22].

However, a few studies linked the macro-level factors (e.g., income level, wealth, education, etc) to hypertension [23–27]. For example, income levels were associated with a reduced chance of achieving blood pressure control [24]. Also, another study found that State socioeconomic indicators may contribute to the burden of hypertension in community-dwelling adults in the US [23]. To address these gaps, we combine the World Happiness Report and the NCD-RisC, combining emotional (subjective well-being) factors such as generosity, freedom, social support, and perceptions of corruption with macro-level socioeconomic indicators (e.g., Gross Domestic Product (GDP), life expectancy), and apply machine learning techniques to examine sex-specific patterns in hypertension prevalence across 151 countries. Understanding the country-specific characteristics that are associated with hypertension at the macro level can be the first step toward developing effective public health policies related to well-being [28–30].

The World Happiness Report (WHR) provides variables explaining variations in national happiness including GDP per capita, generosity, healthy life expectancy, perceptions of corruption, freedom to make life choices, and social support, and also ranks countries based on people’s self-assessed happiness [31]. This study examines the relationship between national happiness and hypertension prevalence across countries and sexes using World Happiness Report (WHR) and NCD (non-communicable disease) Risk Factor Collaboration (NCD-RisC) for the year 2019 [31,32]. We assessed the association between key variables that explain variations in national happiness scores on predicting hypertension prevalence using machine learning, hypothesizing that the factors associated with national happiness may be indirectly associated with hypertension prevalence at the national level. Our research questions focused on whether countries with higher happiness scores tend to have lower hypertension prevalence across sexes, whether these key variables are associated with hypertension prevalence across sexes, and to what degree they contribute to improving the model prediction of this relationship.

## Methods

### Study setting, data, and design

Happiness data were obtained from the WHR, a partnership of Gallup, the Oxford Wellbeing Research Centre, the UN Sustainable Development Solutions Network, and the WHR’s Editorial Board [33–36]. The WHR includes complete data on 151 countries, as well as six variables that may explain variations in national happiness scores: Gross domestic product (GDP) per capita, generosity, healthy life expectancy, perceptions of corruption, freedom to make life choices, and social support (see Table 1) [36]. World Happiness Report measures happiness score (life evaluations), using the 0–10 Cantril Self-Anchoring Striving Scale, in which respondents rate their current happiness (life) from the worst possible (0) to the best possible (10). While the overall happiness score is based on the Cantril Ladder Self-Anchoring Striving Scale as measured in the Gallup World Poll, the six structural factors are utilized to explain and model the variation in these scores between countries. Each happiness feature (variable) represents the weighted contribution of that factor to the total happiness score, illustrating how much each component improves life evaluation relative to a baseline state. We used the WHR contribution components because they represent standardized estimates of the relative contribution of each determinant to life evaluation within the WHR analytical framework, allowing clearer interpretation and comparability across countries. The 2019 WHR scores are based on Gallup World Poll data collected across multiple years and compiled for the 2019 report. The prevalences of hypertension were obtained from NCD-RisC, a global network of health scientists that provides data on risk factors for non-communicable diseases [32]. NCD-RisC reported separate prevalences of hypertension among males and females. Hypertension data for 2019 were collected from the population-representative sample, using pooled data from 1201 studies covering 104 million participants for adults aged 30–79 years, as of the end of 2019 [6]. The final study dataset was created by matching the most recent available hypertension data (2019 NCD-RisC) with the 2019 WHR indicators (based on the 2016–2018 Gallup World Poll averages), using standardized International Organization for Standardization (ISO) 3-alpha country codes.After excluding countries with missing variables, the final sample consisted of a concurrent group of 151 countries (See Table A in the S1 Appendix).

**Table 1:** Sample characteristics of the happiness features and hypertension prevalence in 151 countries.

Variable	Minimum	Median	Mean	Maximum	Standard Deviation
Happiness score	2·85	5·37	5·41	7·77	1·13
Gross Domestic Product (GDP) per capita	0	0·96	0·91	1·68	0·40
Social support	0	1·29	1·21	1·62	0·30
Healthy life expectancy	0	0·79	0·72	1·14	0·24
Freedom to make life choices	0	0·42	0·39	0·63	0·15
Generosity	0	0·18	0·18	0·57	0·10
Perceptions of corruption	0	0·09	0·11	0·45	0·09
Prevalence of Hypertension Male	0·23	0·36	0·38	0·62	0·08
Prevalence of Hypertension Female	0·17	0·36	0·36	0·51	0·08

The happiness score is based on the Cantril Self-Anchoring Striving Scale, where respondents rate their current lives on a scale of 0–10, with 0 being the worst possible life and 10 being the best. Darker green shows a higher Happiness score.

### Statistical analysis

Countries were clustered based on their six contributing features (variables) to happiness using the K-means clustering method. This clustering algorithm divides data into K distinct clusters. K-means clustering finds the center of each cluster based on cluster mean and minimizes the total squared distance between all data points in the cluster and its center [37]. Compared with hierarchical clustering and density-based approaches (e.g., DBSCAN), K-means provides greater stability, computational efficiency, and interpretability when compact clusters and a predefined number of groups are desired. Prior to clustering, all variables were standardized to a zero mean and unit variance to prevent scale-driven dominance in distance calculations. Cluster robustness was assessed by repeating the clustering procedure across multiple initializations and optimization runs, yielding consistent cluster assignments. We used the NbClust package in R, which evaluates multiple clustering validity indices, such as the Hubert and D indices, to determine the best number of clusters in the dataset [38]. The final number of clusters (4) was determined using a majority voting rule, where the value of 4 recommended by the largest number of indices across the evaluated criteria was selected as the optimal clustering solution. Since happiness data have six dimensions to reduce dimension, we used Principal Component Analysis (PCA) to project happiness data onto the first two principal components, which were then used as inputs for clustering; the resulting clusters were visualized in two dimensions [39]. We analyzed country-level hypertension prevalence data separately for males and females based on the clustering of happiness features.

To explore the potential association between contributing features to happiness and hypertension, we used the extreme gradient boosting (XGBoost) machine learning model to predict the sex-stratified hypertension prevalences [40]. XGBoost is a nonparametric, tree‐based regression approach that excels at considering possible nonlinearities, autocorrelation, and multicollinearity in predictive modeling [41,42]. We reported the main results using XGBoost Gain relative importance. The gain reflects the relative influence of individual features on improving the prediction accuracy. In other words, a higher gain indicates that a variable is more influential in the model, because it contributes more to reducing prediction error [43,44]. We also checked for overfitting or selection bias and applied a 10‐fold cross‐validation to tune the model’s hyperparameters and gauge the generalizability of the results [45]. To mitigate overfitting given the modest sample size, we employed cross-validation and leveraged XGBoost’s built-in regularization mechanisms to constrain model complexity. Model hyperparameters were selected to balance predictive performance and generalizability, ensuring robustness in a limited-sample setting. For reproducibility, a fixed random seed was set prior to model training in R to ensure consistent cross-validation splits and model estimation across runs. We also conducted a SHapley Additive exPlanations (SHAP) analysis to provide a more localized view by measuring the positive or negative impact of each feature on Hypertension prevalence [46]. SHAP values were computed using the TreeSHAP algorithm, which provides an efficient and exact estimation of Shapley values for tree-based ensemble models such as XGBoost. Feature importance was evaluated using both Gain and SHAP values, which capture complementary aspects of model behavior. Differences in feature rankings reflect their distinct methodological foundations and are interpreted as complementary perspectives rather than inconsistencies. Because our analysis is based on cross-sectional, ecological data, the observed associations should not be interpreted as causal and may be influenced by unmeasured confounding or ecological bias. We used RStudio 4·0·2 (R Core Team, 2020) for the analysis. All the datasets used in the analysis are aggregated and open-source data, which was deemed as non-human-subject research and, as a result, no IRB approval was needed.

## Results

**Fig 1:**
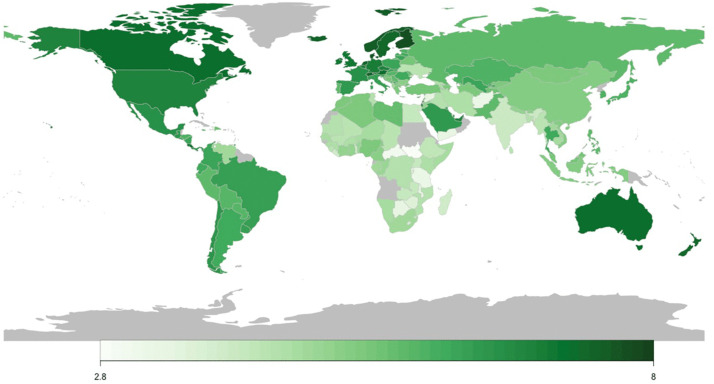
Happiness Score in 2019. The happiness score is based on the Cantril Self-Anchoring Striving Scale, where respondents rate their current lives on a scale of 0 to 10, with 0 being the worst possible life and 10 being the best. Darker green shows a higher Happiness score. Countries with no available data are shown in gray.

Table 1 provides sample characteristics (descriptive statistics) of the happiness features and hypertension prevalence in the 151 countries included in our study. Happiness scores ranged from 2·85 (South Sudan) to 7·77 (Finland), with higher scores indicating a greater level of happiness, and an average score of 5·41 with a standard deviation of 1·13. Fig 1 depicts the Happiness score map in 2019.

Based on the happiness features, the K-means clustering method resulted in four clusters of countries. Table B in S1 Appendix shows the number of indices and measures used to determine the number of clusters in a dataset. We visualized the data using PCA and projected happiness features into the first two principal components, containing the most variation in the data (Figure A in S1 Appendix). A value of 49·7 on the x-axis which shows the first principal component implies that the first principal component (mainly influenced by GDP per capita, healthy life expectancy and social support) accounts for 49·7% of happiness variation. The second principal component (mainly influenced by generosity and perceptions of corruption) on the y-axis accounts for 24·2% of the happiness variation.

**Fig 2:**
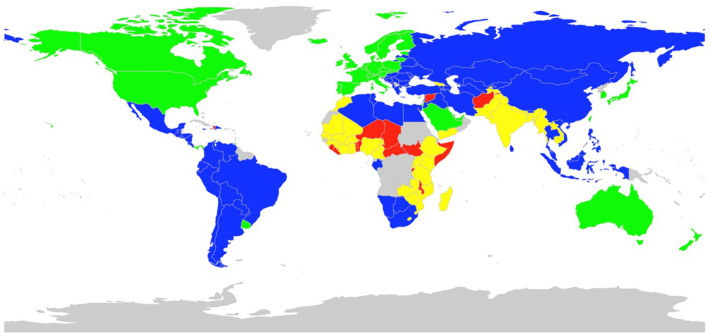
Mapping of clustering results of countries’ happiness features (variables). **Cluster 1 (Green):** Australia, Austria, Bahrain, Belgium, Canada, Cyprus, Czech Republic, Denmark, Estonia, Finland, France, Germany, Iceland, Ireland, Israel, Italy, Japan, Kuwait, Lithuania, Luxembourg, Malta, Netherlands, New Zealand, Norway, Panama, Poland, Portugal, Qatar, Saudi Arabia, Singapore, Slovakia, Slovenia, South Korea, Spain, Sweden, Switzerland, Taiwan, Trinidad and Tobago, United Arab Emirates, United Kingdom, United States of America, Uruguay. **Cluster 2 (Blue):** Albania, Algeria, Argentina, Armenia, Azerbaijan, Belarus, Bhutan, Bolivia, Bosnia and Herzegovina, Botswana, Brazil, Bulgaria, Chile, China, Colombia, Costa Rica, Croatia, Dominican, Republic, Ecuador, Egypt, El Salvador, Gabon, Greece, Guatemala, Honduras, Hungary, Indonesia, Iran, Iraq, Jamaica, Jordan, Kazakhstan, Kyrgyzstan, Latvia, Lebanon, Libya, Macedonia (TFYR), Malaysia, Mauritius, Mexico, Moldova, Mongolia, Montenegro, Namibia, Nicaragua, Palestinian Territories, Paraguay, Peru, Philippines, Romania, Russia, Serbia, South Africa, Sri Lanka, Thailand, Tunisia, Turkey, Turkmenistan, Ukraine, Uzbekistan, Venezuela, Vietnam. **Cluster 3 (Yellow):** Bangladesh, Burkina Faso, Cambodia, Cameroon, Ethiopia, Gambia, Georgia, Ghana, Guinea, India, Ivory Coast, Kenya, Laos, Lesotho, Madagascar, Mali, Mauritania, Morocco, Mozambique, Myanmar, Nepal, Nigeria, Pakistan, Rwanda, Senegal, Swaziland, Tajikistan, Tanzania, Uganda, Yemen, Zambia, Zimbabwe. **Cluster 4 (Red):** Afghanistan, Benin, Burundi, Central African Republic, Chad, Comoros, Haiti, Liberia, Malawi, Niger, Sierra Leone, Somalia, South Sudan, Syria, Togo. Countries with no available data are shown in gray.

Cluster 1 consists of 42 countries, mainly European countries, Japan, South Korea, Taiwan, the United States of America, and Australia. There are also a few Middle Eastern countries (Saudi Arabia, Kuwait, Qatar, and Bahrain) in Cluster 1 (Fig 2). This cluster exhibits a high happiness score (mean: 6·7) and the average hypertension prevalence for males (mean: 0·38) and the lowest hypertension prevalence for females (mean 0·29). Slovenia, Lithuania, and Poland rank among the countries with the highest hypertension prevalence for males.

Cluster 2 consists of 62 countries from South America, Africa, and the Middle East with an average happiness score (mean: 5·40). It has the highest hypertension prevalence for males and higher hypertension prevalence for females (mean: 0·41 and 0·38, respectively). Cluster 3 includes 32 countries in Africa and South Asia and has an average of 4·46, which is lower than the overall happiness average score. This cluster also has the lowest hypertension rates for males (mean: 0·34) and slightly higher to average hypertension rates for females (mean: 0·37). Cluster 4 consists of 15 countries from Africa, in addition to Afghanistan and Syria. This cluster has low happiness scores (mean: 3·9). It also has an average to relatively low hypertension prevalence (mean: 0·35) for males and a higher hypertension prevalence for females (mean: 0·39). Tables D and F in S1 Appendix show the ranking of hypertension prevalence and happiness features by country for males and females.

The results from the evaluation metrics and goodness of fit in the 10‐fold assessment for females (R2 = 0.48, adjusted R2 = 0·48, MAE = 0·04, and RMSE = 0·05) and males (R2 = 0.30, adjusted R2 = 0·28, MAE = 0·05, and RMSE = 0·06) summarize the observed discrepancy between the predicted and actual hypertension prevalence (Table F in S1 Appendix).

**Fig 3:**
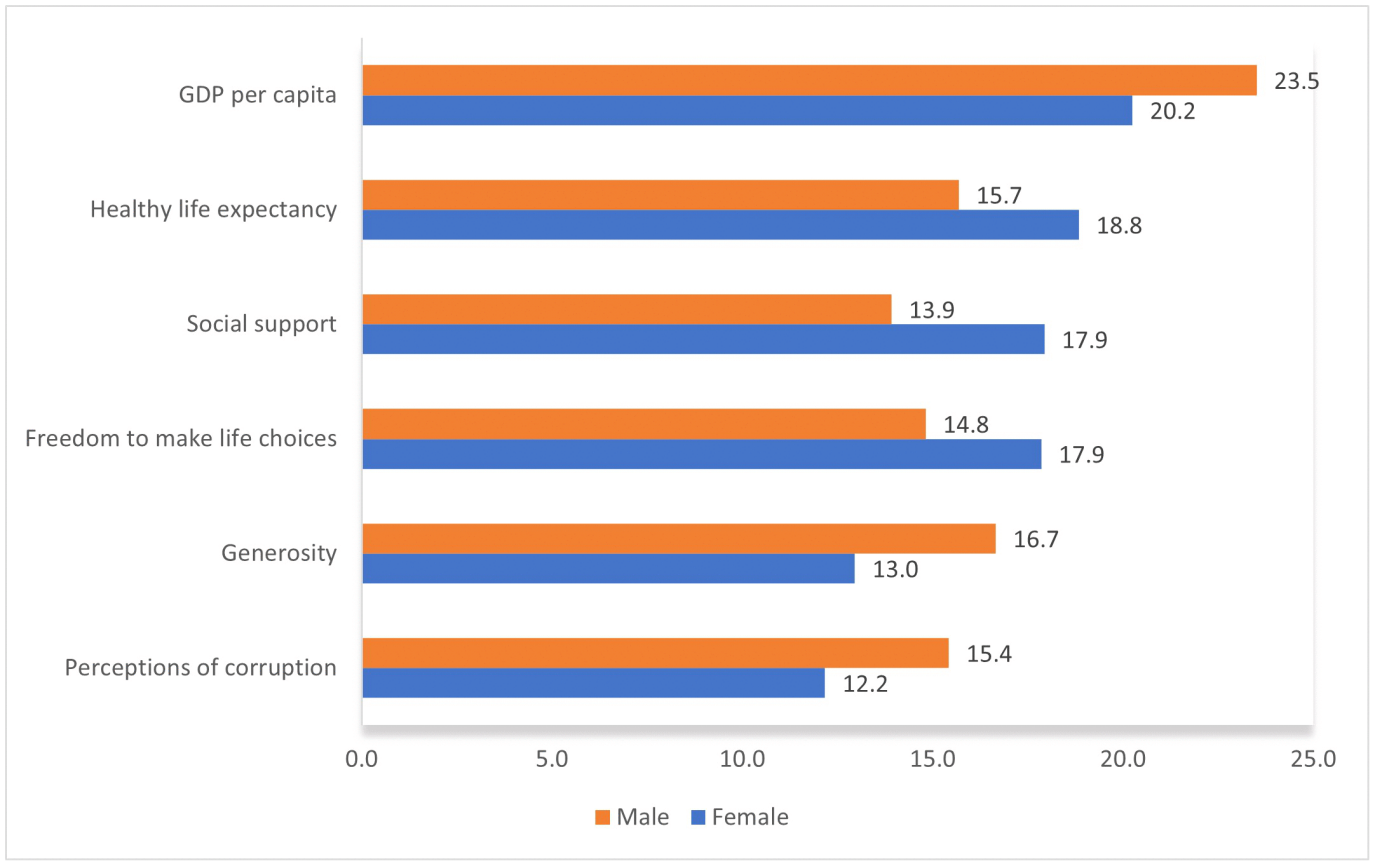
Results of XGBoost Analysis, the Gain Relative Importance of Each Happiness Features (variable). Gain denotes the indicator’s (feature) relative contribution in explaining variation in outcomes (i.e., hypertension prevalence rate). A higher feature gain implies greater importance of the feature for generating a prediction. Feature gains were normalized and presented as percentages summing to 100%.

Fig 3 shows the results of the XGBoost models and depicts the Gain relative importance of each happiness feature in predicting the prevalence of hypertension. GDP per capita in a country is the most important feature in predicting the prevalence of hypertension for males, followed by generosity and life expectancy. The relative contributions of these features are 23·5%, 16·7%, and 15·7%, respectively. Also, perceptions of corruption, freedom to make life choices, and social support have relative contributions of 15·4%, 14·8%, and 13·9%, respectively (see Tables G and H in S1 Appendix). Similarly, GDP per capita with a relative gain of 20·2% has the highest contribution in predicting the prevalence of Hypertension in females. Healthy life expectancy and social support have relative contributions of 18·8% and 17·9%. Freedom to make life choices, generosity, and perception of corruption have relative contributions of 17·9%, 13·0%, and 12·2%, respectively. Tables G and H in S1 Appendix show the feature importance matrix of the prevalence of hypertension and happiness features for females and males.

The results of the SHAP plot, which were used to visualize the features associated with the prevalence of hypertension, show that social support, healthy life expectancy, and freedom to make choices had the most positive SHAP values, indicating that higher values of these predictors contributed to higher predicted hypertension prevalence in the model in males. Also, healthy life expectancy, GDP per capita, and social support had the most significant positive SHAP contributions with the prevalence of hypertension in females (Figures B and C and Table I in the Appendix).

## Discussion

This study aimed to investigate the association between features of happiness and the prevalence of hypertension across 151 countries. By employing both clustering techniques and XGBoost machine learning methods, we sought to identify potential patterns and predictors underlying this association. The clustering analysis shows countries characterized by higher happiness scores tended to exhibit lower rates of hypertension, suggesting a potential inverse relationship between happiness and cardiovascular health. We observed diversity in happiness and health outcomes. Cluster 1, composed predominantly of high-income European countries, exhibited the highest happiness scores and the average prevalence of hypertension for males and the lowest for females. Cluster 2 from South America, Africa, and the Middle East has an average happiness score and the highest hypertension prevalence for males and a higher hypertension prevalence for females. Cluster 3 includes countries in Africa and South Asia and has a lower than the overall happiness average score, and the lowest hypertension prevalence for males and slightly higher hypertension rates for females. Cluster 4, which includes several African countries and conflict-affected regions, reported the lowest happiness scores and lower hypertension prevalence for males and higher hypertension prevalence for females.

Our results are consistent with previous studies in high-income countries, which found that happier nations report systematically lower levels of hypertension and a negative correlation between high blood pressure and life satisfaction [22,47]. This association may reflect lower material stress and greater economic stability characteristic of high-income countries. These factors facilitate improved access to healthcare, healthier living conditions, and preventive services, all of which are consistently associated with better cardiovascular outcomes [48–50].

Clusters 2 and 3 represent countries in transitional stages of the epidemiological shift. The relative importance of modifiable cardiovascular risk factors varies significantly by national income level, with metabolic and lifestyle risks contributing differentially to disease burden across economic contexts [51]. In many middle-income countries, factors such as poor diet and inactive lifestyles account for a substantial share of cardiovascular disease, particularly hypertension, where economic growth has outpaced the reach of local healthcare and preventive services [51].

Cluster 4 consists of countries includes several African and Middle Eastern countries, which are conflict-affected regions with low happiness and moderate hypertension. In conflict-affected countries, health systems often experience significant disruption of preventive and therapeutic services, which may contribute to reduced detection and management of chronic conditions such as hypertension despite the presence of high levels of population stress [52–56]. Additionally, these countries have a lower income, which might cause higher material stress and lower economic stability, lower access to healthcare, and preventive services, which are associated with poor cardiovascular outcomes [48–50]. Also, in lower-income countries, the negative association between income inequality and happiness is driven more by perceptions of unfairness and lack of trust than by income levels themselves [57].

The PCA analysis revealed that GDP per capita, and generosity explain 49·7% and 24·2% of the variation in happiness across countries, respectively. The XGBoost analysis results showed that for males, GDP per capita, with a relative gain of 23·5%, is the most significant factor in predicting hypertension prevalence, followed by generosity and life expectancy with relative gains of 16·7% and 15·7% in males. Also, GDP per capita has the highest predictive contribution for hypertension prevalence with a relative gain of 20·2%, while healthy life expectancy and social support contribute 18·8% and 17·9%, respectively, in females. What is more, “freedom to make life choices” plays a more important role in the model prediction for females than for males. On the other hand, “perceptions of corruption” have more predictive power for males than females.

The relationship between low happiness and high hypertension prevalence suggests a potential pattern of co-occurrence, where poor subjective well-being may be linked to higher blood pressure levels at the macro level. GDP per capita and generosity demonstrate that while wealthier nations tend to score higher in happiness, other factors such as generosity contribute to happiness in lower-income countries. In other words, the significant contribution of generosity to happiness variation suggests that fostering a culture of giving and support may enhance overall well-being, particularly in countries where economic conditions may not be optimal.

Our main findings align with previous research emphasizing the multifaceted benefits of subjective well-being on overall health outcomes, including cardiovascular health [17,19,35]. Although our results are based on country-level associations, prior individual-level research suggests that the mechanisms behind this protective effect may include lower stress levels, better immune function, and healthier lifestyle choices often associated with happier individuals [58,59]. These findings underscore the importance of promoting mental and emotional well-being as a component of public health strategies aimed at reducing the burden of cardiovascular disease [58–61] and establishing well-being as a primary policy objective [29].

Examining details of contributing features to national happiness indicates that each contributing component of happiness may have a different effect on the prevalence of hypertension. This finding underscores the essential role of economic prosperity in shaping population health [61,62], emphasizes the importance of the socioeconomic gradient in hypertension including social support, job characteristics, and socioeconomic status. In addition, the effect of these features might not be the same for different sexes. For example, High strain and active jobs, but not job insecurity, were related to increased cardiovascular disease risk among females [63]. Although GDP per capita, as a proxy for income, shows the most relative gain compared to other components, its importance is higher for males. This may be related to the observation that individuals who struggle to make ends meet and cover basic daily expenses for their families are more likely to develop high blood pressure [59,64].

Generosity and life expectancy are also significant predictors of hypertension prevalence in males while healthy life expectancy and social support emerged as important factors for females. Following GDP per capita, generosity is the second most important predictor of the prevalence of hypertension. The reason may lie in the fact that altruistic behavior can enhance existential well-being outcomes [33,65]. Indeed, prosocial orientation may influence mood and facilitate social integration, divert people from their own problems and stress, and satisfy the need for competence [66–73]. These factors can strengthen cardiovascular and physiological immune responses, which may lead to improvements in health [71].

According to SHAP values, for males, social support, healthy life expectancy, and freedom to make choices have the most positive SHAP contributions, meaning higher values of these variables were associated with higher predicted hypertension prevalence in the model, while for females, healthy life expectancy, GDP per capita, and social support are the top contributors. Hence, based on these two metrics, while GDP per capita is critical in predicting hypertension (as per the gain metric), social factors like social support and life expectancy (highlighted by SHAP) are equally, if not more, influential for individuals’ well-being and health. The inconsistency might reflect the difference between what drives happiness and health at the country level (e.g., GDP per capita) versus the individual level (e.g., social support). This shows that macro-level improvements like increasing GDP might not trickle down to improve individual well-being unless social policies are in place to address individual needs.

The study has several limitations. First, the cross-sectional nature of the data and the reliance on self-reported measures of happiness and a population-representative sample, using pooled data for hypertension prevalence, may lead to measurement bias. Concepts of happiness are culturally shaped; therefore, variations in how respondents interpret and report happiness may influence WHR-reported scores. Our findings should be interpreted as reflecting self-reported life evaluations, which may be subject to cultural interpretation. However, cultural variations in response styles and the conceptualization of well-being can influence happiness scores, and research indicates that these measures are sufficiently robust and remain broadly comparable across countries. [74] Second, non-linear interactions and potential confounding variables not included in the model could influence the results. Although efforts were made to tune the model and apply cross-validation to prevent overfitting, some degree of model bias is inevitable. Exploring relative measures such as relative income and the moderating effects of other cultural, social, and environmental factors on the relationships studied could offer a more comprehensive understanding of global disparities in hypertension prevalence. Also, NCD-RisC estimates are derived from pooled analyses of heterogeneous survey data, which may introduce uncertainty and potential measurement bias. [75] Third, the associations observed may be influenced by reverse causality, where lower hypertension prevalence could contribute to higher healthy life expectancy levels rather than the other way around. Also, our study employs an ecological, cross-sectional design using country-level averages, therefore, a causal relationship or directionality between national happiness and hypertension prevalence cannot be established. Finally, this analysis was focused on the WHR dataset, therefore, we did not adjust for additional country-level variables such as population age structure, obesity, urbanization, or healthcare access. These unmeasured factors may confound the observed associations and should be examined in future studies that integrate WHR indicators with other global health databases.

## Conclusion

We identified several contributing happiness features that were associated with hypertension prevalence. Our results suggest that these features may be differentially associated with hypertension prevalence across males and females. Specifically, GDP per capita emerged as a prominent predictor for both sexes, with a higher relative gain observed for males. For females, healthy life expectancy and social support showed greater predictive weight, while generosity and life expectancy appeared relatively more influential for males. These findings point toward the potential value of further exploring sex-specific public health considerations to better understand patterns of hypertension prevalence. Moreover, they highlight the importance of a multi-faceted, integrated policy approach that considers not only economic factors but also broader societal well-being.

## Supporting information

S1 Appendix Supplementary tables and figures on clustering, happiness, and hypertension prevalence. (DOCX)
