# Supplementary material for: Happiness and hypertension prevalence: A global analysis
Source: PLOS Glob Public Health. 2026 Jun 3;6(6):e0006472. doi: 10.1371/journal.pgph.0006472 (PMC13232796; doi:10.1371/journal.pgph.0006472)
Supplement: S1 Appendix — (DOCX) [file pgph.0006472.s001.docx]

**S1 APPENDIX**


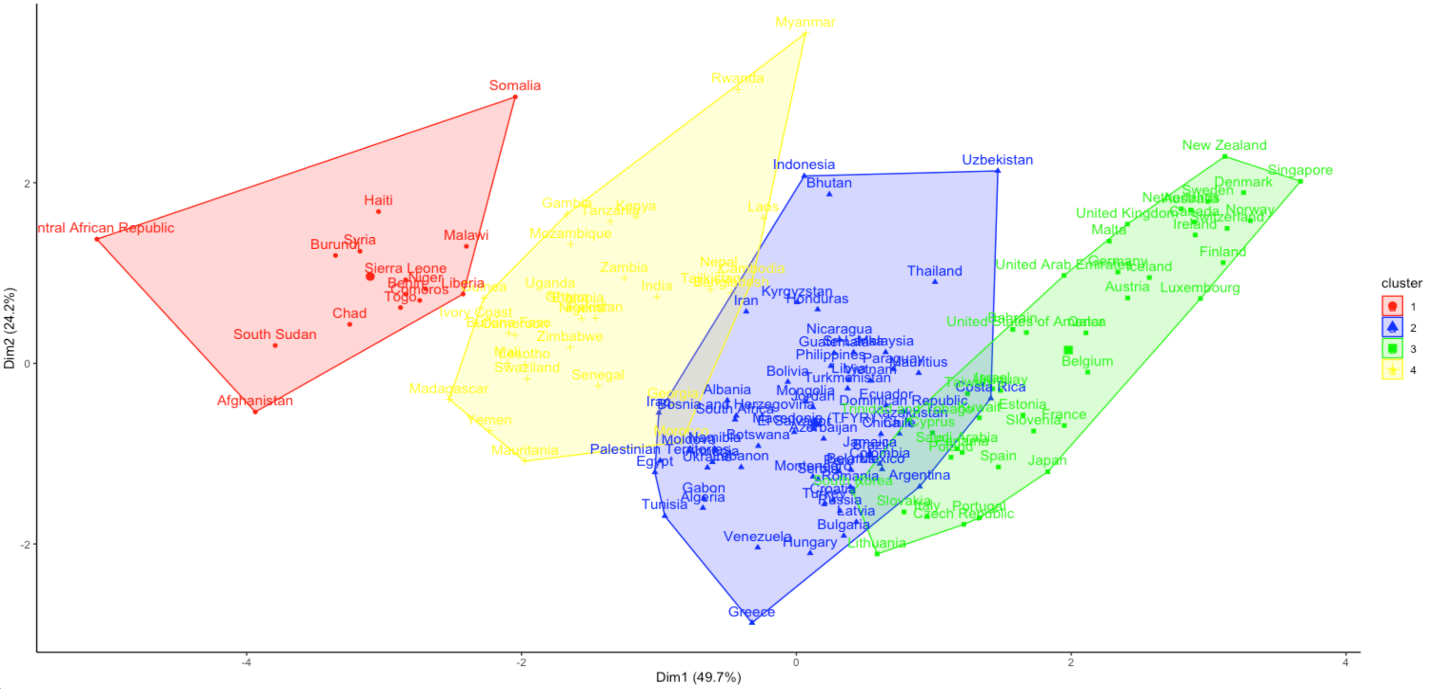


**Figure A:** Clustering Results of Countries' Happiness Features (variables) Using the K-means Clustering Algorithm and Visualized Using Principal Components Analysis

**Cluster 1 (Green):** Australia, Austria, Bahrain, Belgium, Canada, Cyprus, Czech Republic, Denmark, Estonia, Finland, France, Germany, Iceland, Ireland, Israel, Italy, Japan, Kuwait, Lithuania, Luxembourg, Malta, Netherlands, New Zealand, Norway, Panama, Poland, Portugal, Qatar, Saudi Arabia, Singapore, Slovakia, Slovenia, South Korea, Spain, Sweden, Switzerland, Taiwan, Trinidad and Tobago, United Arab Emirates, United Kingdom, United States of America, Uruguay. **Cluster 2 (Blue):** Albania, Algeria, Argentina, Armenia, Azerbaijan, Belarus, Bhutan, Bolivia, Bosnia and Herzegovina, Botswana, Brazil, Bulgaria, Chile, China, Colombia, Costa Rica, Croatia, Dominican, Republic, Ecuador, Egypt, El Salvador, Gabon, Greece, Guatemala, Honduras, Hungary, Indonesia, Iran, Iraq, Jamaica, Jordan, Kazakhstan, Kyrgyzstan, Latvia, Lebanon, Libya, Macedonia (TFYR), Malaysia, Mauritius, Mexico, Moldova, Mongolia, Montenegro, Namibia, Nicaragua, Palestinian Territories, Paraguay, Peru, Philippines, Romania, Russia, Serbia, South Africa, Sri Lanka, Thailand, Tunisia, Turkey, Turkmenistan, Ukraine, Uzbekistan, Venezuela, Vietnam. **Cluster 3 (Yellow):** Bangladesh, Burkina Faso, Cambodia, Cameroon, Ethiopia, Gambia, Georgia, Ghana, Guinea, India, Ivory Coast, Kenya, Laos, Lesotho, Madagascar, Mali, Mauritania, Morocco, Mozambique, Myanmar, Nepal, Nigeria, Pakistan, Rwanda, Senegal, Swaziland, Tajikistan, Tanzania, Uganda, Yemen, Zambia, Zimbabwe. **Cluster 4 (Red):** Afghanistan, Benin, Burundi, Central African Republic, Chad, Comoros, Haiti, Liberia, Malawi, Niger, Sierra Leone, Somalia, South Sudan, Syria, Togo.

The first principal component (PC1), shown on the x-axis, explains 49.7% of the total variance and is primarily influenced by GDP per capita, healthy life expectancy, and social support. The second principal component (PC2), shown on the y-axis, explains 24.2% of the variance and is mainly driven by generosity and perceptions of corruption. Together, these components capture the majority of variation in national happiness scores.

**Figure B:** Results of the SHAP Analysis for Male

**Figure C:** Results of the SHAP Analysis for Female

**Table A:** Definition of Happiness Features (variable)

| **Feature** | **Definition** |  |
| --- | --- | --- |
| **GDP per capita** | The statistics of Gross Domestic Product (GDP) per capita in purchasing power parity (PPP) at constant 2011 international dollar prices. |  |
|  |  |  |
| **Generosity** | Generosity is the residual of regressing the national average of response to the Gallup World Poll (GWP) question “Have you donated money to a charity in the past month?” on GDP per capita. |  |
|  |  |  |
| **Healthy life expectancy** | Based on the World Health Organization’s (WHO) Global Health Observatory data repository, healthy life expectancy is a measure that combines both quantity and quality of life, assessing the average number of years that a person can expect to live in "full health". |  |
|  |  |  |
| **Perceptions of corruption** | Corruption Perception: The measure is the national average of the survey responses to two questions in the Gallup World Poll (GWP): “Is corruption widespread throughout the government or not” and “Is corruption widespread within businesses or not?” level is just the average response of the overall perception at the individual level. |  |
|  |  |  |
|  |  |  |
|  |  |  |
| **Freedom to make life choices** | Freedom to make life choices is the national average of responses to the Gallup World Poll (GWP) question “Are you satisfied or dissatisfied with your freedom to choose what you do with your life?” |  |
|  |  |  |
|  |  |  |
| **Social support** | Social support (or having someone to count on in times of trouble) is the national average of the binary responses (either 0 or 1) to the Gallup World Poll (GWP) question “If you were in trouble, do you have relatives or friends you can count on to help you whenever you need them, or not?” |  |
|  |  |  |
|  |  |  |

**Table B:** The Number of Indices Used to Determine the Number of Clusters in the Dataset

| **Proposed Best Number of Clusters** | **Number of Indices** |
| --- | --- |
| Proposed 3 as the best number of clusters | 3 |
| Proposed 4 as the best number of clusters | 14 |
| Proposed 5 as the best number of clusters | 4 |
| Proposed 8 as the best number of clusters | 1 |
| Proposed 9 as the best number of clusters | 1 |

**Table C:** Clusters and Country Information

| **Cluster** | **Color** | **Countries** |
| --- | --- | --- |
| Cluster 1 | Green | Australia, Austria, Bahrain, Belgium, Canada, Cyprus, Czech Republic, Denmark, Estonia, Finland, France, Germany, Iceland, Ireland, Israel, Italy, Japan, Kuwait, Lithuania, Luxembourg, Malta, Netherlands, New Zealand, Norway, Panama, Poland, Portugal, Qatar, Saudi Arabia, Singapore, Slovakia, Slovenia, South Korea, Spain, Sweden, Switzerland, Taiwan, Trinidad and Tobago, United Arab Emirates, United Kingdom, United States of America, Uruguay. |
| Cluster 2 | Blue | Albania, Algeria, Argentina, Armenia, Azerbaijan, Belarus, Bhutan, Bolivia, Bosnia and Herzegovina, Botswana, Brazil, Bulgaria, Chile, China, Colombia, Costa Rica, Croatia, Dominican Republic, Ecuador, Egypt, El Salvador, Gabon, Greece, Guatemala, Honduras, Hungary, Indonesia, Iran, Iraq, Jamaica, Jordan, Kazakhstan, Kyrgyzstan, Latvia, Lebanon, Libya, Macedonia (TFYR), Malaysia, Mauritius, Mexico, Moldova, Mongolia, Montenegro, Namibia, Nicaragua, Palestinian Territories, Paraguay, Peru, Philippines, Romania, Russia, Serbia, South Africa, Sri Lanka, Thailand, Tunisia, Turkey, Turkmenistan, Ukraine, Uzbekistan, Venezuela, Vietnam. |
| Cluster 3 | Yellow | Bangladesh, Burkina Faso, Cambodia, Cameroon, Ethiopia, Gambia, Georgia, Ghana, Guinea, India, Ivory Coast, Kenya, Laos, Lesotho, Madagascar, Mali, Mauritania, Morocco, Mozambique, Myanmar, Nepal, Nigeria, Pakistan, Rwanda, Senegal, Swaziland, Tajikistan, Tanzania, Uganda, Yemen, Zambia, Zimbabwe. |
| Cluster 4 | Red | Afghanistan, Benin, Burundi, Central African Republic, Chad, Comoros, Haiti, Liberia, Malawi, Niger, Sierra Leone, Somalia, South Sudan, Syria, Togo. |
| No Data | Gray | Countries with no available data. |

**Table D:** Ranking of Prevalence of Hypertension and Happiness Features (variables) by Country for Female in 2019

| **Country** | **Cluster** | **Prevalence of Hypertension for Male** | **Prevalence of Hypertension for Female** | **Happiness score** | **GDP per capita** | **Social support** | **Healthy life expectancy** | **Freedom to make life choices** | **Generosity** | **Perceptions of corruption** |
| --- | --- | --- | --- | --- | --- | --- | --- | --- | --- | --- |
| **Switzerland** | **1** | 0·26 | 0·17 | 7·48 | 1·45 | 1·53 | 1·05 | 0·57 | 0·26 | 0·34 |
| **Peru** | **2** | 0·23 | 0·18 | 5·70 | 0·96 | 1·27 | 0·85 | 0·45 | 0·08 | 0·03 |
| **Canada** | **1** | 0·24 | 0·20 | 7·28 | 1·36 | 1·50 | 1·04 | 0·58 | 0·29 | 0·31 |
| **Taiwan** | **1** | 0·27 | 0·21 | 6·45 | 1·37 | 1·43 | 0·91 | 0·35 | 0·24 | 0·10 |
| **Spain** | **1** | 0·34 | 0·21 | 6·35 | 1·29 | 1·48 | 1·06 | 0·36 | 0·15 | 0·08 |
| **South Korea** | **1** | 0·32 | 0·21 | 5·89 | 1·30 | 1·22 | 1·04 | 0·16 | 0·17 | 0·06 |
| **Japan** | **1** | 0·40 | 0·22 | 5·89 | 1·33 | 1·42 | 1·09 | 0·44 | 0·07 | 0·14 |
| **United Kingdom** | **1** | 0·30 | 0·23 | 7·05 | 1·33 | 1·54 | 1·00 | 0·45 | 0·35 | 0·28 |
| **China** | **2** | 0·30 | 0·24 | 5·19 | 1·03 | 1·12 | 0·89 | 0·52 | 0·06 | 0·10 |
| **Iceland** | **1** | 0·31 | 0·24 | 7·49 | 1·38 | 1·62 | 1·03 | 0·59 | 0·35 | 0·12 |
| **Luxembourg** | **1** | 0·37 | 0·24 | 7·09 | 1·61 | 1·48 | 1·01 | 0·53 | 0·19 | 0·32 |
| **France** | **1** | 0·34 | 0·24 | 6·59 | 1·32 | 1·47 | 1·05 | 0·44 | 0·11 | 0·18 |
| **Sweden** | **1** | 0·36 | 0·25 | 7·34 | 1·39 | 1·49 | 1·01 | 0·57 | 0·27 | 0·37 |
| **Netherlands** | **1** | 0·36 | 0·25 | 7·49 | 1·40 | 1·52 | 1·00 | 0·56 | 0·32 | 0·30 |
| **Malta** | **1** | 0·34 | 0·25 | 6·73 | 1·30 | 1·52 | 1·00 | 0·56 | 0·38 | 0·15 |
| **Germany** | **1** | 0·34 | 0·25 | 6·99 | 1·37 | 1·45 | 0·99 | 0·50 | 0·26 | 0·26 |
| **Ecuador** | **2** | 0·29 | 0·25 | 6·03 | 0·91 | 1·31 | 0·87 | 0·50 | 0·13 | 0·09 |
| **Israel** | **1** | 0·33 | 0·25 | 7·14 | 1·28 | 1·45 | 1·03 | 0·37 | 0·26 | 0·08 |
| **Cambodia** | **3** | 0·26 | 0·25 | 4·70 | 0·57 | 1·12 | 0·64 | 0·61 | 0·23 | 0·06 |
| **Norway** | **1** | 0·35 | 0·25 | 7·55 | 1·49 | 1·58 | 1·03 | 0·60 | 0·27 | 0·34 |
| **Iran** | **2** | 0·27 | 0·26 | 4·55 | 1·10 | 0·84 | 0·79 | 0·30 | 0·27 | 0·12 |
| **Cyprus** | **1** | 0·36 | 0·26 | 6·05 | 1·26 | 1·22 | 1·04 | 0·41 | 0·19 | 0·04 |
| **Belgium** | **1** | 0·34 | 0·26 | 6·92 | 1·36 | 1·50 | 0·99 | 0·47 | 0·16 | 0·21 |
| **Greece** | **2** | 0·37 | 0·26 | 5·29 | 1·18 | 1·16 | 1·00 | 0·07 | 0·00 | 0·03 |
| **Australia** | **1** | 0·32 | 0·26 | 7·23 | 1·37 | 1·55 | 1·04 | 0·56 | 0·33 | 0·29 |
| **Vietnam** | **2** | 0·33 | 0·26 | 5·18 | 0·74 | 1·35 | 0·85 | 0·54 | 0·15 | 0·07 |
| **Ireland** | **1** | 0·38 | 0·27 | 7·02 | 1·50 | 1·55 | 1·00 | 0·52 | 0·30 | 0·31 |
| **Singapore** | **1** | 0·35 | 0·27 | 6·26 | 1·57 | 1·46 | 1·14 | 0·56 | 0·27 | 0·45 |
| **Bolivia** | **2** | 0·29 | 0·27 | 5·78 | 0·78 | 1·21 | 0·71 | 0·51 | 0·14 | 0·06 |
| **New Zealand** | **1** | 0·34 | 0·28 | 7·31 | 1·30 | 1·56 | 1·03 | 0·59 | 0·33 | 0·38 |
| **Portugal** | **1** | 0·37 | 0·28 | 5·69 | 1·22 | 1·43 | 1·00 | 0·51 | 0·05 | 0·02 |
| **Denmark** | **1** | 0·43 | 0·29 | 7·60 | 1·38 | 1·57 | 1·00 | 0·59 | 0·25 | 0·41 |
| **Italy** | **1** | 0·39 | 0·29 | 6·22 | 1·29 | 1·49 | 1·04 | 0·23 | 0·16 | 0·03 |
| **United States of America** | **1** | 0·34 | 0·29 | 6·89 | 1·43 | 1·46 | 0·87 | 0·45 | 0·28 | 0·13 |
| **Thailand** | **2** | 0·29 | 0·29 | 6·01 | 1·05 | 1·41 | 0·83 | 0·56 | 0·36 | 0·03 |
| **Yemen** | **3** | 0·29 | 0·30 | 3·38 | 0·29 | 1·16 | 0·46 | 0·14 | 0·11 | 0·08 |
| **Ethiopia** | **3** | 0·25 | 0·30 | 4·29 | 0·34 | 1·03 | 0·53 | 0·34 | 0·21 | 0·10 |
| **Saudi Arabia** | **1** | 0·36 | 0·30 | 6·37 | 1·40 | 1·36 | 0·80 | 0·44 | 0·08 | 0·13 |
| **Austria** | **1** | 0·37 | 0·30 | 7·25 | 1·38 | 1·48 | 1·02 | 0·53 | 0·24 | 0·23 |
| **India** | **3** | 0·32 | 0·30 | 4·02 | 0·75 | 0·76 | 0·59 | 0·50 | 0·20 | 0·08 |
| **Finland** | **1** | 0·41 | 0·31 | 7·77 | 1·34 | 1·59 | 0·99 | 0·60 | 0·15 | 0·39 |
| **Colombia** | **2** | 0·31 | 0·31 | 6·12 | 0·99 | 1·41 | 0·84 | 0·47 | 0·10 | 0·03 |
| **Rwanda** | **3** | 0·29 | 0·31 | 3·33 | 0·36 | 0·71 | 0·61 | 0·55 | 0·22 | 0·41 |
| **Mexico** | **2** | 0·33 | 0·31 | 6·59 | 1·07 | 1·32 | 0·86 | 0·43 | 0·07 | 0·07 |
| **Laos** | **3** | 0·26 | 0·31 | 4·80 | 0·76 | 1·03 | 0·55 | 0·55 | 0·27 | 0·16 |
| **Malawi** | **4** | 0·27 | 0·32 | 3·41 | 0·19 | 0·56 | 0·50 | 0·44 | 0·22 | 0·09 |
| **Burkina Faso** | **3** | 0·29 | 0·32 | 4·59 | 0·33 | 1·06 | 0·38 | 0·25 | 0·18 | 0·11 |
| **Mauritius** | **2** | 0·34 | 0·32 | 5·89 | 1·12 | 1·40 | 0·80 | 0·50 | 0·22 | 0·06 |
| **Guatemala** | **2** | 0·31 | 0·33 | 6·44 | 0·80 | 1·27 | 0·75 | 0·53 | 0·18 | 0·08 |
| **Philippines** | **2** | 0·35 | 0·33 | 5·63 | 0·81 | 1·29 | 0·66 | 0·56 | 0·12 | 0·11 |
| **Benin** | **4** | 0·29 | 0·33 | 4·88 | 0·39 | 0·44 | 0·40 | 0·35 | 0·18 | 0·08 |
| **Chile** | **2** | 0·39 | 0·33 | 6·44 | 1·16 | 1·37 | 0·92 | 0·36 | 0·19 | 0·06 |
| **El Salvador** | **2** | 0·31 | 0·34 | 6·25 | 0·79 | 1·24 | 0·79 | 0·43 | 0·09 | 0·07 |
| **Zambia** | **3** | 0·30 | 0·34 | 4·11 | 0·58 | 1·06 | 0·43 | 0·43 | 0·25 | 0·09 |
| **Uganda** | **3** | 0·31 | 0·34 | 4·19 | 0·33 | 1·07 | 0·44 | 0·36 | 0·25 | 0·06 |
| **Nepal** | **3** | 0·40 | 0·34 | 4·91 | 0·45 | 1·23 | 0·68 | 0·44 | 0·28 | 0·09 |
| **Czech Republic** | **1** | 0·49 | 0·34 | 6·85 | 1·27 | 1·49 | 0·92 | 0·46 | 0·05 | 0·04 |
| **Estonia** | **1** | 0·46 | 0·34 | 5·89 | 1·24 | 1·53 | 0·87 | 0·50 | 0·10 | 0·16 |
| **Lebanon** | **2** | 0·42 | 0·34 | 5·20 | 0·99 | 1·22 | 0·81 | 0·22 | 0·17 | 0·03 |
| **Bangladesh** | **3** | 0·24 | 0·34 | 4·46 | 0·56 | 0·93 | 0·72 | 0·53 | 0·17 | 0·14 |
| **Turkey** | **2** | 0·31 | 0·34 | 5·37 | 1·18 | 1·36 | 0·81 | 0·19 | 0·08 | 0·11 |
| **Honduras** | **2** | 0·33 | 0·34 | 5·86 | 0·64 | 1·24 | 0·83 | 0·51 | 0·25 | 0·08 |
| **United Arab Emirates** | **1** | 0·44 | 0·34 | 6·82 | 1·50 | 1·31 | 0·82 | 0·60 | 0·26 | 0·18 |
| **Kenya** | **3** | 0·31 | 0·35 | 4·51 | 0·51 | 0·98 | 0·58 | 0·43 | 0·37 | 0·05 |
| **Tunisia** | **2** | 0·34 | 0·35 | 4·46 | 0·92 | 1·00 | 0·81 | 0·17 | 0·06 | 0·05 |
| **Kuwait** | **1** | 0·44 | 0·35 | 6·02 | 1·50 | 1·32 | 0·81 | 0·49 | 0·14 | 0·10 |
| **Panama** | **1** | 0·37 | 0·35 | 6·32 | 1·15 | 1·44 | 0·91 | 0·52 | 0·11 | 0·05 |
| **Bahrain** | **1** | 0·40 | 0·35 | 6·20 | 1·36 | 1·37 | 0·87 | 0·54 | 0·25 | 0·11 |
| **Tanzania** | **3** | 0·31 | 0·35 | 3·23 | 0·48 | 0·88 | 0·50 | 0·42 | 0·28 | 0·15 |
| **Morocco** | **3** | 0·35 | 0·36 | 5·21 | 0·80 | 0·78 | 0·78 | 0·42 | 0·04 | 0·08 |
| **Burundi** | **4** | 0·33 | 0·36 | 3·78 | 0·05 | 0·45 | 0·38 | 0·22 | 0·18 | 0·18 |
| **Jordan** | **2** | 0·40 | 0·36 | 4·91 | 0·84 | 1·22 | 0·81 | 0·38 | 0·11 | 0·13 |
| **South Sudan** | **4** | 0·32 | 0·36 | 2·85 | 0·31 | 0·57 | 0·29 | 0·01 | 0·20 | 0·09 |
| **Ghana** | **3** | 0·31 | 0·36 | 5·00 | 0·61 | 0·87 | 0·49 | 0·38 | 0·24 | 0·04 |
| **Comoros** | **4** | 0·30 | 0·36 | 3·97 | 0·27 | 0·76 | 0·51 | 0·14 | 0·27 | 0·08 |
| **Sri Lanka** | **2** | 0·34 | 0·36 | 4·37 | 0·95 | 1·26 | 0·83 | 0·47 | 0·24 | 0·05 |
| **Nicaragua** | **2** | 0·34 | 0·37 | 6·11 | 0·69 | 1·32 | 0·83 | 0·43 | 0·20 | 0·13 |
| **Palestinian Territories** | **2** | 0·41 | 0·37 | 4·70 | 0·66 | 1·25 | 0·67 | 0·22 | 0·10 | 0·07 |
| **Algeria** | **2** | 0·35 | 0·37 | 5·21 | 1·00 | 1·16 | 0·79 | 0·09 | 0·07 | 0·11 |
| **Togo** | **4** | 0·34 | 0·37 | 4·08 | 0·28 | 0·57 | 0·41 | 0·29 | 0·18 | 0·09 |
| **Ivory Coast** | **3** | 0·37 | 0·38 | 4·94 | 0·57 | 0·81 | 0·23 | 0·35 | 0·15 | 0·09 |
| **Qatar** | **1** | 0·42 | 0·38 | 6·37 | 1·68 | 1·31 | 0·87 | 0·56 | 0·22 | 0·17 |
| **Slovakia** | **1** | 0·47 | 0·38 | 6·20 | 1·25 | 1·50 | 0·88 | 0·33 | 0·12 | 0·01 |
| **Mali** | **3** | 0·30 | 0·38 | 4·39 | 0·38 | 1·10 | 0·31 | 0·33 | 0·15 | 0·05 |
| **Cameroon** | **3** | 0·35 | 0·39 | 5·04 | 0·55 | 0·91 | 0·33 | 0·38 | 0·19 | 0·04 |
| **Somalia** | **4** | 0·33 | 0·39 | 4·67 | 0·00 | 0·70 | 0·27 | 0·56 | 0·24 | 0·27 |
| **Gabon** | **2** | 0·36 | 0·39 | 4·80 | 1·06 | 1·18 | 0·57 | 0·29 | 0·04 | 0·05 |
| **Latvia** | **2** | 0·49 | 0·39 | 5·94 | 1·19 | 1·46 | 0·81 | 0·26 | 0·07 | 0·06 |
| **Uruguay** | **1** | 0·46 | 0·39 | 6·29 | 1·12 | 1·46 | 0·89 | 0·52 | 0·13 | 0·15 |
| **Madagascar** | **3** | 0·35 | 0·39 | 3·93 | 0·27 | 0·92 | 0·55 | 0·15 | 0·17 | 0·04 |
| **Nigeria** | **3** | 0·33 | 0·39 | 5·26 | 0·70 | 1·11 | 0·25 | 0·43 | 0·22 | 0·04 |
| **Venezuela** | **2** | 0·40 | 0·39 | 4·71 | 0·96 | 1·43 | 0·81 | 0·15 | 0·06 | 0·05 |
| **Costa Rica** | **2** | 0·36 | 0·39 | 7·17 | 1·03 | 1·44 | 0·96 | 0·56 | 0·14 | 0·09 |
| **Libya** | **2** | 0·46 | 0·39 | 5·53 | 1·04 | 1·30 | 0·67 | 0·42 | 0·13 | 0·15 |
| **Mauritania** | **3** | 0·36 | 0·40 | 4·49 | 0·57 | 1·17 | 0·49 | 0·07 | 0·11 | 0·09 |
| **Syria** | **4** | 0·42 | 0·40 | 3·46 | 0·62 | 0·38 | 0·44 | 0·01 | 0·33 | 0·14 |
| **Myanmar** | **3** | 0·35 | 0·40 | 4·36 | 0·71 | 1·18 | 0·55 | 0·53 | 0·57 | 0·17 |
| **Turkmenistan** | **2** | 0·38 | 0·40 | 5·25 | 1·05 | 1·54 | 0·66 | 0·39 | 0·24 | 0·03 |
| **Gambia** | **3** | 0·35 | 0·40 | 4·52 | 0·31 | 0·94 | 0·43 | 0·38 | 0·27 | 0·17 |
| **Chad** | **4** | 0·35 | 0·41 | 4·35 | 0·35 | 0·77 | 0·19 | 0·17 | 0·20 | 0·08 |
| **Slovenia** | **1** | 0·50 | 0·41 | 6·12 | 1·26 | 1·52 | 0·95 | 0·56 | 0·14 | 0·06 |
| **Mongolia** | **2** | 0·45 | 0·41 | 5·29 | 0·95 | 1·53 | 0·67 | 0·32 | 0·24 | 0·04 |
| **Macedonia (TFYR)** | **2** | 0·49 | 0·41 | 5·27 | 0·98 | 1·29 | 0·84 | 0·34 | 0·18 | 0·03 |
| **Egypt** | **2** | 0·36 | 0·41 | 4·17 | 0·91 | 1·04 | 0·64 | 0·24 | 0·08 | 0·07 |
| **Bulgaria** | **2** | 0·49 | 0·41 | 5·01 | 1·09 | 1·51 | 0·81 | 0·31 | 0·08 | 0·00 |
| **Albania** | **2** | 0·43 | 0·41 | 4·72 | 0·95 | 0·85 | 0·87 | 0·38 | 0·18 | 0·03 |
| **Montenegro** | **2** | 0·50 | 0·41 | 5·52 | 1·05 | 1·36 | 0·87 | 0·20 | 0·14 | 0·08 |
| **Hungary** | **2** | 0·56 | 0·41 | 5·76 | 1·20 | 1·41 | 0·83 | 0·20 | 0·08 | 0·02 |
| **Malaysia** | **2** | 0·41 | 0·41 | 5·34 | 1·22 | 1·17 | 0·83 | 0·51 | 0·26 | 0·02 |
| **Russia** | **2** | 0·47 | 0·41 | 5·65 | 1·18 | 1·45 | 0·73 | 0·33 | 0·08 | 0·03 |
| **Argentina** | **2** | 0·54 | 0·41 | 6·09 | 1·09 | 1·43 | 0·88 | 0·47 | 0·07 | 0·05 |
| **Liberia** | **4** | 0·37 | 0·41 | 3·98 | 0·07 | 0·92 | 0·44 | 0·37 | 0·23 | 0·03 |
| **Bosnia and Herzegovina** | **2** | 0·47 | 0·41 | 5·39 | 0·95 | 1·21 | 0·84 | 0·21 | 0·26 | 0·01 |
| **Ukraine** | **2** | 0·45 | 0·42 | 4·33 | 0·82 | 1·39 | 0·74 | 0·18 | 0·19 | 0·01 |
| **Trinidad and Tobago** | **1** | 0·43 | 0·42 | 6·19 | 1·23 | 1·48 | 0·71 | 0·49 | 0·19 | 0·02 |
| **Mozambique** | **3** | 0·34 | 0·42 | 4·47 | 0·20 | 0·99 | 0·39 | 0·49 | 0·20 | 0·14 |
| **Lithuania** | **1** | 0·54 | 0·42 | 6·15 | 1·24 | 1·52 | 0·82 | 0·29 | 0·04 | 0·04 |
| **Brazil** | **2** | 0·48 | 0·42 | 6·30 | 1·00 | 1·44 | 0·80 | 0·39 | 0·10 | 0·09 |
| **Serbia** | **2** | 0·50 | 0·42 | 5·60 | 1·00 | 1·38 | 0·85 | 0·28 | 0·14 | 0·04 |
| **Azerbaijan** | **2** | 0·40 | 0·42 | 5·21 | 1·04 | 1·15 | 0·77 | 0·35 | 0·04 | 0·18 |
| **Georgia** | **3** | 0·47 | 0·42 | 4·52 | 0·89 | 0·67 | 0·75 | 0·35 | 0·04 | 0·16 |
| **Guinea** | **3** | 0·38 | 0·42 | 4·53 | 0·38 | 0·83 | 0·38 | 0·33 | 0·21 | 0·09 |
| **Kazakhstan** | **2** | 0·41 | 0·43 | 5·81 | 1·17 | 1·51 | 0·73 | 0·41 | 0·15 | 0·10 |
| **Niger** | **4** | 0·40 | 0·43 | 4·63 | 0·14 | 0·77 | 0·37 | 0·32 | 0·19 | 0·10 |
| **Poland** | **1** | 0·55 | 0·43 | 6·18 | 1·21 | 1·44 | 0·88 | 0·48 | 0·12 | 0·05 |
| **Central African Republic** | **4** | 0·39 | 0·43 | 3·08 | 0·03 | 0·00 | 0·11 | 0·23 | 0·24 | 0·04 |
| **Tajikistan** | **3** | 0·51 | 0·43 | 5·47 | 0·49 | 1·10 | 0·72 | 0·39 | 0·23 | 0·14 |
| **Senegal** | **3** | 0·37 | 0·43 | 4·68 | 0·45 | 1·13 | 0·57 | 0·29 | 0·15 | 0·07 |
| **Bhutan** | **2** | 0·44 | 0·43 | 5·08 | 0·81 | 1·32 | 0·60 | 0·46 | 0·37 | 0·17 |
| **Kyrgyzstan** | **2** | 0·38 | 0·43 | 5·26 | 0·55 | 1·44 | 0·72 | 0·51 | 0·30 | 0·02 |
| **Sierra Leone** | **4** | 0·38 | 0·43 | 4·37 | 0·27 | 0·84 | 0·24 | 0·31 | 0·25 | 0·05 |
| **Romania** | **2** | 0·53 | 0·44 | 6·07 | 1·16 | 1·23 | 0·82 | 0·46 | 0·08 | 0·00 |
| **South Africa** | **2** | 0·44 | 0·44 | 4·72 | 0·96 | 1·35 | 0·47 | 0·39 | 0·13 | 0·05 |
| **Indonesia** | **2** | 0·36 | 0·45 | 5·19 | 0·93 | 1·20 | 0·66 | 0·49 | 0·50 | 0·03 |
| **Uzbekistan** | **2** | 0·47 | 0·45 | 6·17 | 0·75 | 1·53 | 0·76 | 0·63 | 0·32 | 0·24 |
| **Pakistan** | **3** | 0·42 | 0·45 | 5·65 | 0·68 | 0·89 | 0·54 | 0·31 | 0·22 | 0·10 |
| **Namibia** | **2** | 0·43 | 0·45 | 4·64 | 0·88 | 1·31 | 0·48 | 0·40 | 0·07 | 0·06 |
| **Afghanistan** | **4** | 0·35 | 0·45 | 3·20 | 0·35 | 0·52 | 0·36 | 0·00 | 0·16 | 0·02 |
| **Croatia** | **2** | 0·51 | 0·45 | 5·43 | 1·15 | 1·27 | 0·91 | 0·30 | 0·12 | 0·02 |
| **Armenia** | **2** | 0·48 | 0·46 | 4·56 | 0·85 | 1·05 | 0·81 | 0·28 | 0·10 | 0·06 |
| **Zimbabwe** | **3** | 0·37 | 0·46 | 3·66 | 0·37 | 1·11 | 0·43 | 0·36 | 0·15 | 0·09 |
| **Belarus** | **2** | 0·52 | 0·47 | 5·32 | 1·07 | 1·47 | 0·79 | 0·23 | 0·09 | 0·14 |
| **Lesotho** | **3** | 0·32 | 0·47 | 3·80 | 0·49 | 1·17 | 0·17 | 0·36 | 0·11 | 0·09 |
| **Moldova** | **2** | 0·49 | 0·47 | 5·53 | 0·69 | 1·33 | 0·74 | 0·25 | 0·18 | 0·00 |
| **Botswana** | **2** | 0·40 | 0·47 | 3·49 | 1·04 | 1·15 | 0·54 | 0·46 | 0·02 | 0·10 |
| **Swaziland** | **3** | 0·37 | 0·47 | 4·21 | 0·81 | 1·15 | 0·00 | 0·31 | 0·07 | 0·13 |
| **Iraq** | **2** | 0·48 | 0·48 | 4·44 | 1·04 | 0·98 | 0·57 | 0·24 | 0·15 | 0·09 |
| **Haiti** | **4** | 0·38 | 0·48 | 3·60 | 0·32 | 0·69 | 0·45 | 0·03 | 0·42 | 0·11 |
| **Jamaica** | **2** | 0·45 | 0·48 | 5·89 | 0·83 | 1·48 | 0·83 | 0·49 | 0·11 | 0·03 |
| **Dominican Republic** | **2** | 0·49 | 0·49 | 5·43 | 1·02 | 1·40 | 0·78 | 0·50 | 0·11 | 0·10 |
| **Paraguay** | **2** | 0·62 | 0·51 | 5·74 | 0·86 | 1·48 | 0·78 | 0·51 | 0·18 | 0·08 |

**Table E:** Ranking of Prevalence of Hypertension and Happiness Features (variables) by Country for Male in 2019

| **Country** | **Cluster** | **Prevalence of Hypertension for Male** | **Prevalence of Hypertension for Female** | **Happiness score** | **GDP per capita** | **Social support** | **Healthy life expectancy** | **Freedom to make life choices** | **Generosity** | **Perceptions of corruption** |
| --- | --- | --- | --- | --- | --- | --- | --- | --- | --- | --- |
| **Peru** | **2** | 0·23 | 0·18 | 5·70 | 0·96 | 1·27 | 0·85 | 0·45 | 0·08 | 0·03 |
| **Bangladesh** | **3** | 0·24 | 0·34 | 4·46 | 0·56 | 0·93 | 0·72 | 0·53 | 0·17 | 0·14 |
| **Canada** | **1** | 0·24 | 0·20 | 7·28 | 1·36 | 1·50 | 1·04 | 0·58 | 0·29 | 0·31 |
| **Ethiopia** | **3** | 0·25 | 0·30 | 4·29 | 0·34 | 1·03 | 0·53 | 0·34 | 0·21 | 0·10 |
| **Laos** | **3** | 0·26 | 0·31 | 4·80 | 0·76 | 1·03 | 0·55 | 0·55 | 0·27 | 0·16 |
| **Cambodia** | **3** | 0·26 | 0·25 | 4·70 | 0·57 | 1·12 | 0·64 | 0·61 | 0·23 | 0·06 |
| **Switzerland** | **1** | 0·26 | 0·17 | 7·48 | 1·45 | 1·53 | 1·05 | 0·57 | 0·26 | 0·34 |
| **Iran** | **2** | 0·27 | 0·26 | 4·55 | 1·10 | 0·84 | 0·79 | 0·30 | 0·27 | 0·12 |
| **Malawi** | **4** | 0·27 | 0·32 | 3·41 | 0·19 | 0·56 | 0·50 | 0·44 | 0·22 | 0·09 |
| **Taiwan** | **1** | 0·27 | 0·21 | 6·45 | 1·37 | 1·43 | 0·91 | 0·35 | 0·24 | 0·10 |
| **Rwanda** | **3** | 0·29 | 0·31 | 3·33 | 0·36 | 0·71 | 0·61 | 0·55 | 0·22 | 0·41 |
| **Benin** | **4** | 0·29 | 0·33 | 4·88 | 0·39 | 0·44 | 0·40 | 0·35 | 0·18 | 0·08 |
| **Yemen** | **3** | 0·29 | 0·30 | 3·38 | 0·29 | 1·16 | 0·46 | 0·14 | 0·11 | 0·08 |
| **Burkina Faso** | **3** | 0·29 | 0·32 | 4·59 | 0·33 | 1·06 | 0·38 | 0·25 | 0·18 | 0·11 |
| **Thailand** | **2** | 0·29 | 0·29 | 6·01 | 1·05 | 1·41 | 0·83 | 0·56 | 0·36 | 0·03 |
| **Ecuador** | **2** | 0·29 | 0·25 | 6·03 | 0·91 | 1·31 | 0·87 | 0·50 | 0·13 | 0·09 |
| **Bolivia** | **2** | 0·29 | 0·27 | 5·78 | 0·78 | 1·21 | 0·71 | 0·51 | 0·14 | 0·06 |
| **United Kingdom** | **1** | 0·30 | 0·23 | 7·05 | 1·33 | 1·54 | 1·00 | 0·45 | 0·35 | 0·28 |
| **Comoros** | **4** | 0·30 | 0·36 | 3·97 | 0·27 | 0·76 | 0·51 | 0·14 | 0·27 | 0·08 |
| **China** | **2** | 0·30 | 0·24 | 5·19 | 1·03 | 1·12 | 0·89 | 0·52 | 0·06 | 0·10 |
| **Zambia** | **3** | 0·30 | 0·34 | 4·11 | 0·58 | 1·06 | 0·43 | 0·43 | 0·25 | 0·09 |
| **Mali** | **3** | 0·30 | 0·38 | 4·39 | 0·38 | 1·10 | 0·31 | 0·33 | 0·15 | 0·05 |
| **Turkey** | **2** | 0·31 | 0·34 | 5·37 | 1·18 | 1·36 | 0·81 | 0·19 | 0·08 | 0·11 |
| **Uganda** | **3** | 0·31 | 0·34 | 4·19 | 0·33 | 1·07 | 0·44 | 0·36 | 0·25 | 0·06 |
| **Iceland** | **1** | 0·31 | 0·24 | 7·49 | 1·38 | 1·62 | 1·03 | 0·59 | 0·35 | 0·12 |
| **Tanzania** | **3** | 0·31 | 0·35 | 3·23 | 0·48 | 0·88 | 0·50 | 0·42 | 0·28 | 0·15 |
| **Colombia** | **2** | 0·31 | 0·31 | 6·12 | 0·99 | 1·41 | 0·84 | 0·47 | 0·10 | 0·03 |
| **El Salvador** | **2** | 0·31 | 0·34 | 6·25 | 0·79 | 1·24 | 0·79 | 0·43 | 0·09 | 0·07 |
| **Kenya** | **3** | 0·31 | 0·35 | 4·51 | 0·51 | 0·98 | 0·58 | 0·43 | 0·37 | 0·05 |
| **Ghana** | **3** | 0·31 | 0·36 | 5·00 | 0·61 | 0·87 | 0·49 | 0·38 | 0·24 | 0·04 |
| **Guatemala** | **2** | 0·31 | 0·33 | 6·44 | 0·80 | 1·27 | 0·75 | 0·53 | 0·18 | 0·08 |
| **India** | **3** | 0·32 | 0·30 | 4·02 | 0·75 | 0·76 | 0·59 | 0·50 | 0·20 | 0·08 |
| **South Korea** | **1** | 0·32 | 0·21 | 5·89 | 1·30 | 1·22 | 1·04 | 0·16 | 0·17 | 0·06 |
| **Lesotho** | **3** | 0·32 | 0·47 | 3·80 | 0·49 | 1·17 | 0·17 | 0·36 | 0·11 | 0·09 |
| **Australia** | **1** | 0·32 | 0·26 | 7·23 | 1·37 | 1·55 | 1·04 | 0·56 | 0·33 | 0·29 |
| **South Sudan** | **4** | 0·32 | 0·36 | 2·85 | 0·31 | 0·57 | 0·29 | 0·01 | 0·20 | 0·09 |
| **Burundi** | **4** | 0·33 | 0·36 | 3·78 | 0·05 | 0·45 | 0·38 | 0·22 | 0·18 | 0·18 |
| **Mexico** | **2** | 0·33 | 0·31 | 6·59 | 1·07 | 1·32 | 0·86 | 0·43 | 0·07 | 0·07 |
| **Vietnam** | **2** | 0·33 | 0·26 | 5·18 | 0·74 | 1·35 | 0·85 | 0·54 | 0·15 | 0·07 |
| **Israel** | **1** | 0·33 | 0·25 | 7·14 | 1·28 | 1·45 | 1·03 | 0·37 | 0·26 | 0·08 |
| **Nigeria** | **3** | 0·33 | 0·39 | 5·26 | 0·70 | 1·11 | 0·25 | 0·43 | 0·22 | 0·04 |
| **Honduras** | **2** | 0·33 | 0·34 | 5·86 | 0·64 | 1·24 | 0·83 | 0·51 | 0·25 | 0·08 |
| **Somalia** | **4** | 0·33 | 0·39 | 4·67 | 0·00 | 0·70 | 0·27 | 0·56 | 0·24 | 0·27 |
| **Spain** | **1** | 0·34 | 0·21 | 6·35 | 1·29 | 1·48 | 1·06 | 0·36 | 0·15 | 0·08 |
| **Belgium** | **1** | 0·34 | 0·26 | 6·92 | 1·36 | 1·50 | 0·99 | 0·47 | 0·16 | 0·21 |
| **Malta** | **1** | 0·34 | 0·25 | 6·73 | 1·30 | 1·52 | 1·00 | 0·56 | 0·38 | 0·15 |
| **United States of America** | **1** | 0·34 | 0·29 | 6·89 | 1·43 | 1·46 | 0·87 | 0·45 | 0·28 | 0·13 |
| **France** | **1** | 0·34 | 0·24 | 6·59 | 1·32 | 1·47 | 1·05 | 0·44 | 0·11 | 0·18 |
| **Mauritius** | **2** | 0·34 | 0·32 | 5·89 | 1·12 | 1·40 | 0·80 | 0·50 | 0·22 | 0·06 |
| **Togo** | **4** | 0·34 | 0·37 | 4·08 | 0·28 | 0·57 | 0·41 | 0·29 | 0·18 | 0·09 |
| **Mozambique** | **3** | 0·34 | 0·42 | 4·47 | 0·20 | 0·99 | 0·39 | 0·49 | 0·20 | 0·14 |
| **Germany** | **1** | 0·34 | 0·25 | 6·99 | 1·37 | 1·45 | 0·99 | 0·50 | 0·26 | 0·26 |
| **New Zealand** | **1** | 0·34 | 0·28 | 7·31 | 1·30 | 1·56 | 1·03 | 0·59 | 0·33 | 0·38 |
| **Sri Lanka** | **2** | 0·34 | 0·36 | 4·37 | 0·95 | 1·26 | 0·83 | 0·47 | 0·24 | 0·05 |
| **Tunisia** | **2** | 0·34 | 0·35 | 4·46 | 0·92 | 1·00 | 0·81 | 0·17 | 0·06 | 0·05 |
| **Nicaragua** | **2** | 0·34 | 0·37 | 6·11 | 0·69 | 1·32 | 0·83 | 0·43 | 0·20 | 0·13 |
| **Philippines** | **2** | 0·35 | 0·33 | 5·63 | 0·81 | 1·29 | 0·66 | 0·56 | 0·12 | 0·11 |
| **Gambia** | **3** | 0·35 | 0·40 | 4·52 | 0·31 | 0·94 | 0·43 | 0·38 | 0·27 | 0·17 |
| **Madagascar** | **3** | 0·35 | 0·39 | 3·93 | 0·27 | 0·92 | 0·55 | 0·15 | 0·17 | 0·04 |
| **Cameroon** | **3** | 0·35 | 0·39 | 5·04 | 0·55 | 0·91 | 0·33 | 0·38 | 0·19 | 0·04 |
| **Algeria** | **2** | 0·35 | 0·37 | 5·21 | 1·00 | 1·16 | 0·79 | 0·09 | 0·07 | 0·11 |
| **Morocco** | **3** | 0·35 | 0·36 | 5·21 | 0·80 | 0·78 | 0·78 | 0·42 | 0·04 | 0·08 |
| **Chad** | **4** | 0·35 | 0·41 | 4·35 | 0·35 | 0·77 | 0·19 | 0·17 | 0·20 | 0·08 |
| **Myanmar** | **3** | 0·35 | 0·40 | 4·36 | 0·71 | 1·18 | 0·55 | 0·53 | 0·57 | 0·17 |
| **Afghanistan** | **4** | 0·35 | 0·45 | 3·20 | 0·35 | 0·52 | 0·36 | 0·00 | 0·16 | 0·02 |
| **Norway** | **1** | 0·35 | 0·25 | 7·55 | 1·49 | 1·58 | 1·03 | 0·60 | 0·27 | 0·34 |
| **Singapore** | **1** | 0·35 | 0·27 | 6·26 | 1·57 | 1·46 | 1·14 | 0·56 | 0·27 | 0·45 |
| **Egypt** | **2** | 0·36 | 0·41 | 4·17 | 0·91 | 1·04 | 0·64 | 0·24 | 0·08 | 0·07 |
| **Sweden** | **1** | 0·36 | 0·25 | 7·34 | 1·39 | 1·49 | 1·01 | 0·57 | 0·27 | 0·37 |
| **Cyprus** | **1** | 0·36 | 0·26 | 6·05 | 1·26 | 1·22 | 1·04 | 0·41 | 0·19 | 0·04 |
| **Indonesia** | **2** | 0·36 | 0·45 | 5·19 | 0·93 | 1·20 | 0·66 | 0·49 | 0·50 | 0·03 |
| **Mauritania** | **3** | 0·36 | 0·40 | 4·49 | 0·57 | 1·17 | 0·49 | 0·07 | 0·11 | 0·09 |
| **Costa Rica** | **2** | 0·36 | 0·39 | 7·17 | 1·03 | 1·44 | 0·96 | 0·56 | 0·14 | 0·09 |
| **Gabon** | **2** | 0·36 | 0·39 | 4·80 | 1·06 | 1·18 | 0·57 | 0·29 | 0·04 | 0·05 |
| **Netherlands** | **1** | 0·36 | 0·25 | 7·49 | 1·40 | 1·52 | 1·00 | 0·56 | 0·32 | 0·30 |
| **Saudi Arabia** | **1** | 0·36 | 0·30 | 6·37 | 1·40 | 1·36 | 0·80 | 0·44 | 0·08 | 0·13 |
| **Greece** | **2** | 0·37 | 0·26 | 5·29 | 1·18 | 1·16 | 1·00 | 0·07 | 0·00 | 0·03 |
| **Luxembourg** | **1** | 0·37 | 0·24 | 7·09 | 1·61 | 1·48 | 1·01 | 0·53 | 0·19 | 0·32 |
| **Panama** | **1** | 0·37 | 0·35 | 6·32 | 1·15 | 1·44 | 0·91 | 0·52 | 0·11 | 0·05 |
| **Zimbabwe** | **3** | 0·37 | 0·46 | 3·66 | 0·37 | 1·11 | 0·43 | 0·36 | 0·15 | 0·09 |
| **Ivory Coast** | **3** | 0·37 | 0·38 | 4·94 | 0·57 | 0·81 | 0·23 | 0·35 | 0·15 | 0·09 |
| **Portugal** | **1** | 0·37 | 0·28 | 5·69 | 1·22 | 1·43 | 1·00 | 0·51 | 0·05 | 0·02 |
| **Senegal** | **3** | 0·37 | 0·43 | 4·68 | 0·45 | 1·13 | 0·57 | 0·29 | 0·15 | 0·07 |
| **Swaziland** | **3** | 0·37 | 0·47 | 4·21 | 0·81 | 1·15 | 0·00 | 0·31 | 0·07 | 0·13 |
| **Liberia** | **4** | 0·37 | 0·41 | 3·98 | 0·07 | 0·92 | 0·44 | 0·37 | 0·23 | 0·03 |
| **Austria** | **1** | 0·37 | 0·30 | 7·25 | 1·38 | 1·48 | 1·02 | 0·53 | 0·24 | 0·23 |
| **Haiti** | **4** | 0·38 | 0·48 | 3·60 | 0·32 | 0·69 | 0·45 | 0·03 | 0·42 | 0·11 |
| **Turkmenistan** | **2** | 0·38 | 0·40 | 5·25 | 1·05 | 1·54 | 0·66 | 0·39 | 0·24 | 0·03 |
| **Sierra Leone** | **4** | 0·38 | 0·43 | 4·37 | 0·27 | 0·84 | 0·24 | 0·31 | 0·25 | 0·05 |
| **Kyrgyzstan** | **2** | 0·38 | 0·43 | 5·26 | 0·55 | 1·44 | 0·72 | 0·51 | 0·30 | 0·02 |
| **Ireland** | **1** | 0·38 | 0·27 | 7·02 | 1·50 | 1·55 | 1·00 | 0·52 | 0·30 | 0·31 |
| **Guinea** | **3** | 0·38 | 0·42 | 4·53 | 0·38 | 0·83 | 0·38 | 0·33 | 0·21 | 0·09 |
| **Chile** | **2** | 0·39 | 0·33 | 6·44 | 1·16 | 1·37 | 0·92 | 0·36 | 0·19 | 0·06 |
| **Italy** | **1** | 0·39 | 0·29 | 6·22 | 1·29 | 1·49 | 1·04 | 0·23 | 0·16 | 0·03 |
| **Central African Republic** | **4** | 0·39 | 0·43 | 3·08 | 0·03 | 0·00 | 0·11 | 0·23 | 0·24 | 0·04 |
| **Jordan** | **2** | 0·40 | 0·36 | 4·91 | 0·84 | 1·22 | 0·81 | 0·38 | 0·11 | 0·13 |
| **Azerbaijan** | **2** | 0·40 | 0·42 | 5·21 | 1·04 | 1·15 | 0·77 | 0·35 | 0·04 | 0·18 |
| **Nepal** | **3** | 0·40 | 0·34 | 4·91 | 0·45 | 1·23 | 0·68 | 0·44 | 0·28 | 0·09 |
| **Venezuela** | **2** | 0·40 | 0·39 | 4·71 | 0·96 | 1·43 | 0·81 | 0·15 | 0·06 | 0·05 |
| **Bahrain** | **1** | 0·40 | 0·35 | 6·20 | 1·36 | 1·37 | 0·87 | 0·54 | 0·25 | 0·11 |
| **Japan** | **1** | 0·40 | 0·22 | 5·89 | 1·33 | 1·42 | 1·09 | 0·44 | 0·07 | 0·14 |
| **Botswana** | **2** | 0·40 | 0·47 | 3·49 | 1·04 | 1·15 | 0·54 | 0·46 | 0·02 | 0·10 |
| **Niger** | **4** | 0·40 | 0·43 | 4·63 | 0·14 | 0·77 | 0·37 | 0·32 | 0·19 | 0·10 |
| **Malaysia** | **2** | 0·41 | 0·41 | 5·34 | 1·22 | 1·17 | 0·83 | 0·51 | 0·26 | 0·02 |
| **Palestinian Territories** | **2** | 0·41 | 0·37 | 4·70 | 0·66 | 1·25 | 0·67 | 0·22 | 0·10 | 0·07 |
| **Kazakhstan** | **2** | 0·41 | 0·43 | 5·81 | 1·17 | 1·51 | 0·73 | 0·41 | 0·15 | 0·10 |
| **Finland** | **1** | 0·41 | 0·31 | 7·77 | 1·34 | 1·59 | 0·99 | 0·60 | 0·15 | 0·39 |
| **Qatar** | **1** | 0·42 | 0·38 | 6·37 | 1·68 | 1·31 | 0·87 | 0·56 | 0·22 | 0·17 |
| **Pakistan** | **3** | 0·42 | 0·45 | 5·65 | 0·68 | 0·89 | 0·54 | 0·31 | 0·22 | 0·10 |
| **Lebanon** | **2** | 0·42 | 0·34 | 5·20 | 0·99 | 1·22 | 0·81 | 0·22 | 0·17 | 0·03 |
| **Syria** | **4** | 0·42 | 0·40 | 3·46 | 0·62 | 0·38 | 0·44 | 0·01 | 0·33 | 0·14 |
| **Albania** | **2** | 0·43 | 0·41 | 4·72 | 0·95 | 0·85 | 0·87 | 0·38 | 0·18 | 0·03 |
| **Namibia** | **2** | 0·43 | 0·45 | 4·64 | 0·88 | 1·31 | 0·48 | 0·40 | 0·07 | 0·06 |
| **Trinidad and Tobago** | **1** | 0·43 | 0·42 | 6·19 | 1·23 | 1·48 | 0·71 | 0·49 | 0·19 | 0·02 |
| **Denmark** | **1** | 0·43 | 0·29 | 7·60 | 1·38 | 1·57 | 1·00 | 0·59 | 0·25 | 0·41 |
| **Kuwait** | **1** | 0·44 | 0·35 | 6·02 | 1·50 | 1·32 | 0·81 | 0·49 | 0·14 | 0·10 |
| **Bhutan** | **2** | 0·44 | 0·43 | 5·08 | 0·81 | 1·32 | 0·60 | 0·46 | 0·37 | 0·17 |
| **South Africa** | **2** | 0·44 | 0·44 | 4·72 | 0·96 | 1·35 | 0·47 | 0·39 | 0·13 | 0·05 |
| **United Arab Emirates** | **1** | 0·44 | 0·34 | 6·82 | 1·50 | 1·31 | 0·82 | 0·60 | 0·26 | 0·18 |
| **Jamaica** | **2** | 0·45 | 0·48 | 5·89 | 0·83 | 1·48 | 0·83 | 0·49 | 0·11 | 0·03 |
| **Ukraine** | **2** | 0·45 | 0·42 | 4·33 | 0·82 | 1·39 | 0·74 | 0·18 | 0·19 | 0·01 |
| **Mongolia** | **2** | 0·45 | 0·41 | 5·29 | 0·95 | 1·53 | 0·67 | 0·32 | 0·24 | 0·04 |
| **Estonia** | **1** | 0·46 | 0·34 | 5·89 | 1·24 | 1·53 | 0·87 | 0·50 | 0·10 | 0·16 |
| **Libya** | **2** | 0·46 | 0·39 | 5·53 | 1·04 | 1·30 | 0·67 | 0·42 | 0·13 | 0·15 |
| **Uruguay** | **1** | 0·46 | 0·39 | 6·29 | 1·12 | 1·46 | 0·89 | 0·52 | 0·13 | 0·15 |
| **Georgia** | **3** | 0·47 | 0·42 | 4·52 | 0·89 | 0·67 | 0·75 | 0·35 | 0·04 | 0·16 |
| **Uzbekistan** | **2** | 0·47 | 0·45 | 6·17 | 0·75 | 1·53 | 0·76 | 0·63 | 0·32 | 0·24 |
| **Bosnia and Herzegovina** | **2** | 0·47 | 0·41 | 5·39 | 0·95 | 1·21 | 0·84 | 0·21 | 0·26 | 0·01 |
| **Russia** | **2** | 0·47 | 0·41 | 5·65 | 1·18 | 1·45 | 0·73 | 0·33 | 0·08 | 0·03 |
| **Slovakia** | **1** | 0·47 | 0·38 | 6·20 | 1·25 | 1·50 | 0·88 | 0·33 | 0·12 | 0·01 |
| **Brazil** | **2** | 0·48 | 0·42 | 6·30 | 1·00 | 1·44 | 0·80 | 0·39 | 0·10 | 0·09 |
| **Iraq** | **2** | 0·48 | 0·48 | 4·44 | 1·04 | 0·98 | 0·57 | 0·24 | 0·15 | 0·09 |
| **Armenia** | **2** | 0·48 | 0·46 | 4·56 | 0·85 | 1·05 | 0·81 | 0·28 | 0·10 | 0·06 |
| **Latvia** | **2** | 0·49 | 0·39 | 5·94 | 1·19 | 1·46 | 0·81 | 0·26 | 0·07 | 0·06 |
| **Dominican Republic** | **2** | 0·49 | 0·49 | 5·43 | 1·02 | 1·40 | 0·78 | 0·50 | 0·11 | 0·10 |
| **Czech Republic** | **1** | 0·49 | 0·34 | 6·85 | 1·27 | 1·49 | 0·92 | 0·46 | 0·05 | 0·04 |
| **Moldova** | **2** | 0·49 | 0·47 | 5·53 | 0·69 | 1·33 | 0·74 | 0·25 | 0·18 | 0·00 |
| **Macedonia (TFYR)** | **2** | 0·49 | 0·41 | 5·27 | 0·98 | 1·29 | 0·84 | 0·34 | 0·18 | 0·03 |
| **Bulgaria** | **2** | 0·49 | 0·41 | 5·01 | 1·09 | 1·51 | 0·81 | 0·31 | 0·08 | 0·00 |
| **Montenegro** | **2** | 0·50 | 0·41 | 5·52 | 1·05 | 1·36 | 0·87 | 0·20 | 0·14 | 0·08 |
| **Slovenia** | **1** | 0·50 | 0·41 | 6·12 | 1·26 | 1·52 | 0·95 | 0·56 | 0·14 | 0·06 |
| **Serbia** | **2** | 0·50 | 0·42 | 5·60 | 1·00 | 1·38 | 0·85 | 0·28 | 0·14 | 0·04 |
| **Tajikistan** | **3** | 0·51 | 0·43 | 5·47 | 0·49 | 1·10 | 0·72 | 0·39 | 0·23 | 0·14 |
| **Croatia** | **2** | 0·51 | 0·45 | 5·43 | 1·15 | 1·27 | 0·91 | 0·30 | 0·12 | 0·02 |
| **Belarus** | **2** | 0·52 | 0·47 | 5·32 | 1·07 | 1·47 | 0·79 | 0·23 | 0·09 | 0·14 |
| **Romania** | **2** | 0·53 | 0·44 | 6·07 | 1·16 | 1·23 | 0·82 | 0·46 | 0·08 | 0·00 |
| **Lithuania** | **1** | 0·54 | 0·42 | 6·15 | 1·24 | 1·52 | 0·82 | 0·29 | 0·04 | 0·04 |
| **Argentina** | **2** | 0·54 | 0·41 | 6·09 | 1·09 | 1·43 | 0·88 | 0·47 | 0·07 | 0·05 |
| **Poland** | **1** | 0·55 | 0·43 | 6·18 | 1·21 | 1·44 | 0·88 | 0·48 | 0·12 | 0·05 |
| **Hungary** | **2** | 0·56 | 0·41 | 5·76 | 1·20 | 1·41 | 0·83 | 0·20 | 0·08 | 0·02 |
| **Paraguay** | **2** | 0·62 | 0·51 | 5·74 | 0·86 | 1·48 | 0·78 | 0·51 | 0·18 | 0·08 |

World Happiness Report measures happiness score (life evaluations), using the 0–10 Cantril Self-Anchoring Striving Scale, in which respondents rate their current happiness (life) from the worst possible (0) to the best possible (10). Each happiness feature (variable) represents the weighted contribution of that factor to the total happiness score, illustrating how much each component improves life evaluation relative to a baseline state.

**Table F:** Goodness of Fit and Prediction Evaluation for the XGBoost Model

| **Measure** | **Female** |  | **Male** |
| --- | --- | --- | --- |
| MAE | 0·043 |  | 0·050 |
| MSE | 0·003 |  | 0·004 |
| RMSE | 0·055 |  | 0·063 |
| R-Squared | 0·483 |  | 0·304 |
| Adjusted R-Squared | 0·465 |  | 0·280 |

**Table G:** Feature Importance Matrix of Prevalence of Hypertension and Happiness Features (variable) for Female in 151 Countries in 2019

| **Feature** | **Gain (%)** | **Cover (%)** | **Frequency (%)** |
| --- | --- | --- | --- |
| GDP per capita | 20·2 | 17·5 | 17·3 |
| Healthy life expectancy | 18·8 | 18·1 | 17·0 |
| Social support | 17·9 | 17·0 | 17·9 |
| Freedom to make life choices | 17·9 | 15·2 | 15·5 |
| Generosity | 13·0 | 16·2 | 16·6 |
| Perceptions of corruption | 12·2 | 16·1 | 15·7 |

XGBoost uses different importance metrics, including gain, cover, and frequency of features. Gain denotes the relative contribution of a feature in the model (i.e., a higher feature gain implies higher importance for generating the prediction). Cover indicates the average coverage of splits that use a specific feature. It corresponds to the percentage of the used observations of feature to decide the leaf node for them. Frequency represents the relative number of times a particular feature occurs across all the trees estimated within the model. Feature importance were normalized and presented as percentages summing to 100%.

**Table H:** Feature Importance Matrix of Prevalence of Hypertension and Happiness Features (variables) for Male in 151 Countries in 2019

| **Feature** | **Gain (%)** | **Cover (%)** | **Frequency (%)** |
| --- | --- | --- | --- |
| GDP per capita | 23·5 | 14·9 | 14·9 |
| Generosity | 16·7 | 22·4 | 21·3 |
| Healthy life expectancy | 15·7 | 16·2 | 17·0 |
| Perceptions of corruption | 15·4 | 15·1 | 15·5 |
| Freedom to make life choices | 14·8 | 16·4 | 16·6 |
| Social support | 13·9 | 15·1 | 14·8 |

XGBoost uses different importance metrics, including gain, cover, and frequency of features. Gain denotes the relative contribution of a feature in the model (i.e., a higher feature gain implies higher importance for generating the prediction). Cover indicates the average coverage of splits that use a specific feature. It corresponds to the percentage of the used observations of feature to decide the leaf node for them. Frequency represents the relative number of times a particular feature occurs across all the trees estimated within the model. Feature importance were normalized and presented as percentages summing to 100%.

**Table I:** Results of the SHAP Analysis for Male and Female

| **Feature** | **Male** | **Female** |
| --- | --- | --- |
| Social support | 0·029 | 0·016 |
| Healthy life expectancy | 0·025 | 0·028 |
| Freedom to make life choices | 0·021 | 0·015 |
| GDP per capita | 0·018 | 0·021 |
| Generosity | 0·018 | 0·011 |
| Perceptions of corruption | 0·013 | 0·012 |

Values represent the mean absolute SHAP contribution of each feature to model predictions, with higher values indicating greater influence on predicted happiness scores.

**S1 Text. Description of K-means clustering and XGBoost (Extreme Gradient Boosting)**

**K-means clustering**

K-means is an unsupervised clustering algorithm that partitions observations into K clusters by minimizing the within-cluster sum of squared distances between observations and their assigned cluster centroids (Hartigan & Wong, 1979). The basic algorithm consists of repeating the following: (1) initializing K centroids, (2) assigning each observation to the nearest centroid, (3) updating each centroid as the mean of observations assigned to that cluster, and (4) repeating steps (2)–(3) until assignments no longer change or a maximum iteration limit is reached. Since K-means can converge to local minima depending on initialization, the algorithm is normally run with multiple random starts and the solution with the lowest within-cluster sum of squares is maintained (Hartigan & Wong, 1979). To avoid one variable dominating the distance calculations when variables are on different scales, it is common to apply standardization before clustering.

**XGBoost (Extreme Gradient Boosting) method**

XGBoost is a supervised learning algorithm based on gradient-boosted decision trees (Chen & Guestrin, 2016). It constructs an additive ensemble of trees sequentially, where each new tree is trained to reduce the errors of the current ensemble by optimizing a differentiable loss function. XGBoost includes regularization terms that penalize model complexity, which helps decrease overfitting and improve generalization (Chen & Guestrin, 2016). Regularly tuned hyperparameters include the number of boosting iterations, learning rate, maximum tree depth, minimum loss reduction, row subsampling, and column subsampling (Chen & Guestrin, 2016). Model development and hyperparameter selection can be conducted using K-fold cross-validation. The dataset is split into K folds, the model is trained on K−1 folds and evaluated on the held-out fold. This process is repeated across all folds. The performance can be summarized using regression metrics such as RMSE and MAE.

**References**

1. Hartigan JA, Wong MA. Algorithm AS 136: A k-means clustering algorithm. *Journal of the Royal Statistical Society: Series C (Applied Statistics).* 1979;28(1):100–108.

Chen T, Guestrin C. XGBoost: A scalable tree boosting system. In: *Proceedings of the 22nd ACM SIGKDD International Conference on Knowledge Discovery and Data Mining.* 2016.”
